# Clinical performance of the Roche Cobas 4800 HPV test for primary cervical cancer screening in a Chinese population

**DOI:** 10.1371/journal.pone.0272721

**Published:** 2022-08-05

**Authors:** Stephanie S. Liu, Karen K. L. Chan, Tina N. Wei, Ka Yu Tse, Siew F. Ngu, Mandy M. Y. Chu, Lesley S. K. Lau, Annie N. Y. Cheung, Hextan Y. S. Ngan

**Affiliations:** 1 Department of Obstetrics & Gynaecology, Li Ka Shing Faculty of Medicine, The University of Hong Kong, Hong Kong SAR, China; 2 Department of Pathology, Li Ka Shing Faculty of Medicine, The University of Hong Kong, Hong Kong SAR, China; Public Library of Science, UNITED KINGDOM

## Abstract

High-risk human papillomavirus (HR-HPV) testing has become an increasing important strategy in primary cervical cancer screening in recent years. It warrants the evaluation of molecular-based HPV tests for accuracy and efficacy of screening. The performance of Roche Cobas 4800 HPV test was validated and compared with Digene Hybrid Capture 2 (HC2) high-risk HPV DNA test for primary screening in a large Chinese screening cohort. Of 6345 women screened, overall agreement between Cobas and HC2 was 92.23% (95% CI: 91.57–92.89). The inter-assay agreement was correlated with the severity of underlying biology, with an increasing concordance found in samples with more severe abnormalities. Most of the discordant samples had the test signal strength closer to the test limits of the detection than concordant samples, reflecting a low viral load and infection of a cluster of low-risk HPV in these samples. The Cobas test demonstrated significantly higher specificity in identifying CIN2+/CIN3+ cases than HC2 test (66.46% vs 43.67% and 65.42% vs 42.86%, p<0.001), with comparable sensitivity in clinical evaluation. Increased specificity of Cobas test would accent women having the highest risk of developing CIN2+, with the potential to reduce unnecessary colposcopy referral in a screening population.

## Introduction

Cervical cancer is a preventable disease and its incidence and associated mortality rate have substantially declined since the implementation of massive screening programs [1]. As HPV has been recognized as a causal agent for cervical cancer [2], molecular techniques to detect the nucleic acid of virus, especially those HR-HPV genotypes, have demonstrated greater accuracy for identifying cervical precancerous lesions and cancer than cytology-based methods in cervical screening [3, 4]. Evidences from number of studies have indicated that replacing cytology-based screening by HPV-based screening can improve the effectiveness of screening, as HPV testing has higher sensitivity relative to Pap test for detection of cervical precancerous lesions and allows screening intervals to be extended up to five years or longer [4–7]. The major limitation of HPV testing is its inability to distinguish transient infections from clinically relevant infections, resulting in reduced specificity compared to cytology, subsequently to high numbers of unnecessary colposcopy referrals [8, 9]. Increase of colposcopy referrals would lead to overscreening and overtreatment of women with transient and/or non-progressing infections.

Currently, several HR-HPV assays have been validated and approved by United States Food and Drug Administration (FDA) [10]. Roche Cobas 4800 HPV test (Roche Molecular Systems, Pleasanton, USA) detects HPV16 and HPV18 genotypes in addition to 12 other HR-HPVs, as additional genotyping could provide more clinical information on the risk of cancer development. It is the first HPV test approved by FDA as a standalone test for primary cervical cancer screening. The clinical sensitivity and specificity of the Cobas HPV test has previously been demonstrated to be comparable with the HC2 test (Qiagen, Germantown MD, USA) [11–16]. However, most of such large comparison studies have been performed in Western countries. In our recent randomized controlled trials (RCT) in Hong Kong, the HC2 test and liquid-based cytology were utilized to compare the screening strategies: co-testing (HPV and cytology) versus cytology alone in the detection of high-grade cervical intraepithelial neoplasia (CIN) for primary cervical screening in a large Chinese population cohort [17]. The present study was a sub-study of the previous RCT study, and the main objective was to compare the performance of the Cobas HPV test to HC2 test for the detection of HR-HPV prevalence and high-grade CIN in a screening cohort. The Linear Array (LA) HPV test was also performed to confirm concordant and resolve discordant Cobas and HC2 results with individual genotype analysis.

## Materials and methods

### Study population and design

The study population was 6345 women who had both HC2 and Cobas HPV tests done were selected from a recent prospectively randomized controlled trial (RCT) in Hong Kong [17]. Briefly, the RCT was a population-based, comparing co-testing of HPV-cytology and cytology alone for the detection of high-grade CIN in primary cervical cancer screening during the period between May 2010 and April 2014. It was registered at ClinicalTrials.gov (NCT01058460) and approved by institutional review board of the University of Hong Kong/Hospital Authority Hong Kong West Cluster (IRB HKU/HA HKWC, No. 09–377) and Kowloon West Cluster (KWC-REC No. KW/EX-13-013(59–14)). Informed written consents were obtained from all the participants. The RTC was comprised of 15,955 women aged 30–60 year old attending routine cervical screening in Hong Kong. Participants were randomized on 1:1 ratio into either the intervention group (co-testing) or the control group (cytology test only). The PreservCyt® Liquid-based cytology (LBC) (Hologic Inc, MA, USA) and HC2 HPV test were performed for all women at the first round screening and only cytology test was done for the follow up screening. Women in the intervention group were managed according to their HPV and cytology results: HC2 positive or LBC above Atypical Squamous Cells of Undetermined Significance (ASCUS) were referred immediately to colposcopy. Women in control group were managed according to cytology results only (HPV result concealed except for triaging of ASCUS): LBC >ASCUS, ASCUS and HPV positive or two consecutive ASCUS results were referred to colposcopy. At colposcopy, four-quadrant biopsies at 3, 6, 9 and 12 o’ clock, and biopsies from the most suspicious areas, as well as endocervical curettage were taken for histological analysis. Women with normal baseline screening results would be invited to attend a follow up screening in 36 months.

### Laboratory tests

Cervical specimens were collected in PreservCyt® for liquid-based cytology, then followed by HC2 test. The HC2 test is a signal amplification assay that utilizes microplate chemiluminescent to detect 13 HR-HPVs. The cutoff (CO) values for each run were calculated based on the relative light units (RLUs) of the positive and negative controls and the results were reported as relative light unit to cutoff (RLU/CO) ratios.

The Cobas test is a real-time PCR based assay targeting HR-HPV L1 gene and human β-globin gene as an internal control. It provides HPV16 and HPV18 genotyping and reports 12 other HR-HPVs as a pooled result. It is a fully automated system from DNA extraction to PCR amplification of HR-HPV DNA and carried out according to manufacturer’s instructions.

The Linear Array HPV genotyping test (LA) generates individual qualitative results for 37 HPV genotypes, including the same 14 HR-HPV genotypes as Cobas test and 23 additional HPV genotypes which are defined as low risk-HPV (LR-HPV) [18]. LA test was performed using the residual PreservCyt fluid from Cobas test and carried out according to manufacturer’s protocol. Specimens positive for at least one HR-HPV genotype were classified as HR-HPV positive.

The results of HC2 HPV test were used for subject management in RCT, whereas the results of Cobas and LA tests were used for comparison analysis only.

### Statistical analysis

All statistical analyses were conducted using computer software SPSS (version 28.0, IBM Corp., Armonk, NY) / GraphPad Prism (version 6) / R (version 3.4.3, R foundation). Overall agreement, positive and negative agreements and corresponding Kappa coefficients with 95% confidence intervals were calculated to estimate levels of agreement between HPV tests. Kappa values from 0 to 0.20, 0.21 to 0.40, 0.41 to 0.60, 0.61 to 0.80 and above 0.81 indicate poor, fair, moderate, good and excellent strength of agreement, respectively. The Median score and Mann-Whitney U tests were used to calculate p values for the median cycle threshold (Ct) values of Cobas and RLU/CO (a relative light unit/cutoff) ratio of HC2 test for concordant versus discordant HR-HPV positive cases.

Specimens that tested positive by Cobas and LA tests for HR-HPV were further analyzed by comparing genotyping results in the following categories: HPV16, HPV18, and other HR HPV types (OHR), as well as coinfections involving the three categories. Samples that were positive for 12 OHR types by Cobas test were considered concordant if the LA provided a HR-HPV genotype (other than HPV 16 and HPV 18), regardless of the number of genotypes detected.

The clinical performance of both Cobas and HC2 tests were analyzed by comparing the sensitivity, specificity, positive and negative predictive values of the test for the detection of CIN2+ and CIN3+, respectively. The unconditional logistic regression model was used to calculate the odds ratio(s) (OR) and 95% confidence interval (s) (CI) for the detection rate of the co-testing group compared to cytology alone group. P values less than 0.05 (two‐sided) were considered statistically significant.

## Results

### Characteristics of the study cohort

The present study included 6345 women with valid cytology and HPV (including HC2 and Cobas tests) results. The mean age was 49.33 years (SD 7.78). A total of 6259 (98.64%) women had normal cytology, 48 (0.76%) had ASCUS and 36 (0.57%) and 2 (0.03%) had low-grade and high-grade intraepithelial lesions (LSIL and HSIL). Two hundred and sixty-one and 241 women were referred to colposcopy from intervention and control groups based on the referral criteria of the corresponding groups.

### HPV prevalence and concordance of Cobas and HC2 tests

Among 6345 women enrolled, Cobas and HC2 tests identified 555 (8.75%) and 506 (7.97%) cervical samples as HR-HPV positive, respectively (Table 1). Of the positive and negative samples identified, 284 and 5568 were detected positive and negative by both tests, respectively. The positive and negative agreements were 53.53% (95% CI: 52.87–54.19) and 95.76% (95% CI: 95.10–96.42). The overall agreement between Cobas and HC2 tests was 92.23% (95% CI: 91.57–92.89) and the Cohen’s kappa coefficient was 0.493 (95% CI: 0.454–0.534, *p* = 0.031), which indicated a moderate agreement. In addition, the Cobas genotyping demonstrated 1.15% (73 of 6345) of HPV16, 0.61% (39 of 6345) of HPV18 and 6.98% (443 of 6345) of 12 OHR HPV positive.

**Table 1:** HPV prevalence and concordance analysis of Cobas and HC2 tests stratified by cytology.

Cytology (No. %)	Cobas+ (%)	HC2+ (%)	Overall agreement (95% CI)	Kappa (95% CI)	*P* value*
Normal (6259, 98.64)	509 (8.13)	457 (7.30)	92.27% (91.61–92.93)	0.457 (0.417–0.497)	0.020
ASCUS (48, 0.76)	20 (41.67)	20 (41.67)	91.67% (83.85–99.49)	0.829 (0.639–0.963)	1.000
LSIL (36, 0.57)	24 (66.67)	27 (75.0)	86.11% (74.81–97.41)	0.667 (0.351–0.898)	0.375
HSIL (2, 0.03)	2 (100)	2 (100)	100%		
Total (6345)	555 (8.75)	506 (7.97)	92.23% (91.57–92.89)	0.493 (0.454–0.534)	0.031

Abbreviations: ASCUS, atypical squamous cells of undetermined significance; LSIL, low-grade squamous intraepithelial lesion; HSIL, high-grade squamous intraepithelial lesion; HR-HPV, high-risk HPV; OHR: other high-risk HPV genotypes; CI, confidence interval.

*: McNemar test.

The HPV prevalence in different cytology groups showed no significant discrepancy of the two tests in HR-HPV detection in disease groups (*p* = 1.0 and *p* = 0.375 in ASCUS and LSIL). Contrary, the significant discrepancy of the two test results was found in women with normal cytology (*p* = 0.02).

The HPV viral load signal was compared in concordant and discordant cases by analysis of the Ct value of Cobas and the RLU/CO ratio of HC2 test (Table 2). Concordant Cobas+/HC2+ samples had significantly lower median Cobas Ct values than discordant Cobas+/HC2- samples (32.10 vs 39.10, *p*<0.001). Similarly, Concordant Cobas+/HC2+ samples had significantly higher median RLU/CO ratio of HC2 than discordant Cobas-/HC2+ samples (33.16 vs 3.61, *p*<0.001). The median Ct value showed the similar patterns when stratified by Cobas genotypes. In addition, the median Ct value of Cobas test in samples of disease groups were significantly lower than samples in normal cytology group (27.90 vs 37.90, *p*<0.001), as well as when stratified by HPV16 and 12 OHR, except HPV18. Likewise, the median RLU/CO ratio of HC2 test was significantly higher in disease group than in normal cytology group (189.23 vs 7.30, *p*<0.001).

**Table 2:** RLU/CO and Ct values for concordant and discordant cases.

	RLU/CO N, Median (Q1, Q3)	Ct (All) N, Median (Q1, Q3)	Ct (12 OHR) N, Median (Q1, Q3)	Ct (HPV16) N, Median (Q1, Q3)	Ct (HPV18) N, Median (Q1, Q3)
Cobas+/HC2+	284, 33.16 (6.52, 208.87)	284, 32.10 (27.40, 36.50)	234, 32.15 (27.55, 36.60)	31, 33.50 (27.80, 36.90)	19, 30.01 (25.60, 33.20)
Cobas-/HC2+	222, 3.61 (1.93, 11.30)	---	---	---	---
Cobas+/HC2-	271, 0.27 (0.18, 0.44)	271, 39.10 (38.20, 39.70)	209, 39.0 (38.15, 39.70)	42, 39.53 (38.85, 39.94)	20, 38.60 (37.10, 39.58)
Cobas-/HC2-	5568, 0.2 (0.15, 0.26)	---	---	---	---

Abbreviations: RLU/CO, relative light unit/cutoff; Ct: cycle threshold; Q1 first quartile; Q3 third quartile; N, number of cases.

### Correlation of Cobas and HC2 results with Linear Array

Linear Array HPV genotyping test was performed on 687 cases to confirm the results of Cobas and HC2 tests. HR-HPV positivity was confirmed in 92.14% (258 of 280) of concordant Cobas+/HC2+ cases (Table 3). For those with discordant results, Cobas+/HC2- samples were more likely to contain HR-HPV genotypes than Cobas-/HC2+ samples (33.96% vs 13.85%, *p*<0.001). LA test identified LR-HPV genotypes in 24.62% (32 of 130) Cobas-/HC2+ samples, which was significantly more than those detected in discordant Cobas+/HC2- samples (7.46%, 20/268, *p*<0.001). HPV53 and HPV62 were the most commonly found LR-HPV genotypes in discordant Cobas-/HC2+ cases (13 of 23) and Cobas+/HC2- cases (5 of 20), respectively.

**Table 3:** Confirmatory of concordant and discordant results of Cobas and HC2 tests by Linear Array test.

Linear Array (687)	Cobas+ / HC2+ (280)	Cobas+ / HC2- (268)	Cobas- / HC2+ (130)	Cobas- / HC2- (8)
HR-HPV (367)	258 (92.14%)	91 (33.96%)*	18 (13.85%)*	--
HPV16 (33)	23 (8.21%)	10 (3.73%)	--	--
HPV18 (26)	21 (7.50%)	5 (1.87%)	--	--
12 OHR (308)	214 (76.43%)	76 (28.36%)	--	--
LR-HPV (57)	5 (1.79%)	20 (7.46%)^#^	32 (24.62%)^#^	--
Negative (263)	17 (6.07%)	157 (58.58%)	80 (61.54%)	8 (100%)

Abbreviations: HR-HPV, high-risk HPV; LR-HPV, low-risk HPV; OHR, other high-risk HPV genotypes.

*: 33.96% vs 13.85%, *p*<0.001.

#: 7.46% vs 24.62%, *p*<0.001.

Of the Cobas genotype identified, LA test detected 43.66% (31/71) of HPV16, 56.41% (22/39) of HPV18 and 64.24% (282/439) of 12 OHR HPV positive. The overall genotyping agreement between Cobas and LA were ranged from 73.36% of 12 OHR, 93.89% of HPV16+ to 96.94% of HPV18+. Only two CIN3 cases were found HPV16+ by both Cobas and LA tests, and HPV18 was negative in all CIN2+ cases.

### Clinical performance of Cobas and HC2 tests in identifying high-grade CIN

Totally 502 cases were referred to colposcopy and cervical and endocervical biopsies were taken for histological assessment. The clinical performance of the Cobas and HC2 tests was evaluated on these cases by comparing the abilities of the tests to detect CIN2+ and CIN3+. CIN2+ lesions were identified in 28 women, while CIN3+ lesions were found in 19 women, including one squamous cell carcinoma (SCC). Table 4 shows the HPV prevalence in the histology groups detected by Cobas and HC2 tests. The overall agreement between two tests was 74.30% (95% CI: 70.48–78.13) and the Cohen’s κ efficient was 0.509 (95% CI: 0.445–0.577, *p*<0.001), indicating a moderate agreement. There was no significant difference in HR-HPV detection between two tests in CIN2+ or CIN3+ samples (*p* = 0.250 and *p* = 0.5). The discrepancy of the two tests was found in women with normal histology or low-grade lesions. HC2 test showed more HR-HPV positive cases than Cobas test in these two groups (54.84% vs 27.96%, and 57.29% vs 37.15%, *p*<0.001).

**Table 4:** HPV prevalence and concordance analysis of Cobas and HC2 tests stratified by histology.

Histology (N)	Cobas				HC 2	Overall agreement (95% CI)	Kappa (95% CI)	*P* value*
	HPV16+ (%)	HPV18+ (%)	12 OHR+ (%)	HR-HPV+ (%)	HR-HPV+ (%)			
Normal (186)	5 (2.69)	6 (3.23)	41 (22.04)	52 (27.96)	102 (54.84)	70.97% (64.44–77.49)	0.443 (0.342–0.560)	<0.001
CIN1 (288)	12 (4.17)	9 (3.13)	86 (29.86)	107 (37.15)	165 (57.29)	75% (70.0–80.0)	0.518 (0.428–0.608)	<0.001
CIN2+ (28)	2 (7.14)	0	21 (75.0)	23 (82.14)	26 (92.86)	89.29% (77.83–100.74)	0.523 (0.0–1.0)	0.250
CIN3+ (19)	2 (10.53)	0	13 (68.42)	15 (78.95)	17 (89.47)	89.47% (75.67–103.27)	0.612 (0.0–1.0)	0.500
Overall (502)	19 (3.78)	15 (2.99)	148 (29.48)	182 (36.25)	293 (58.37)	74.30% (70.48–78.13)	0.509 (0.445–0.577)	<0.001

Abbreviations: N, number of cases; CIN, cervical intraepithelial neoplasia; CIN2+, CIN2 and worse; CIN3+, CIN3 and worse; CI, confidence interval.

*: McNemar test.

The agreements between positive Cobas test and CIN2+/CIN3+ were 67.33% and 65.94%, which were significantly better than the agreement between positive HC2 test and CIN2+/CIN3+ (46.41% and 44.62%, *p*<0.001, Table 5). Cobas test showed a significantly higher specificity than HC2 test in the detection of CIN2+ and CIN3+ (66.46% vs 43.67% and 65.42% vs 42.86%, *p*<0.001). Though HC2 test showed a better sensitivity than Cobas test (92.86% vs 82.14% and 89.47% vs 78.95%), it’s not statistically different (*p* = 0.083 and *p* = 0.157). The sensitivity of HPV16 in the detection of CIN2+ and CIN3+ were very low (7.14% and 10.53%) in our study, whereas its specificity was particularly high (96.41% and 96.48%).

**Table 5:** Clinical performance of Cobas and HC2 tests for detection of CIN2+ and CIN3+ lesions.

HPV test		Agreement* (95%CI)	Sensitivity^#^ (95%CI)	Specificity^$^ (95%CI)	PPV (95%CI)	NPV (95%CI)
Cobas	CIN2+	67.33 (63.23–71.43)	82.14 (77.26–87.03)	66.46 (64.99–67.92)	12.64 (10.98–14.30)	98.44 (97.97–98.91)
	CIN3+	65.94 (61.79–70.08)	78.95 (72.64–85.26)	65.42 (63.97–66.88)	8.24 (6.87–9.62)	98.75 (98.33–99.17)
HC2	CIN2+	46.41 (42.05–50.78)	92.86 (89.57–96.14)	43.67 (42.13–45.21)	8.87 (7.75–9.99)	99.04 (98.59–99.50)
	CIN3+	44.62 (40.27–48.97)	89.47 (84.73–94.22)	42.86 (41.34–44.38)	5.80 (4.88–6.72)	99.04 (98.59–99.50)
HPV16 only (Cobas)	CIN2+	91.43 (88.99–93.88)	7.14 (3.86–10.43)	96.41 (95.84–96.99)	10.53 (5.78–15.28)	94.62 (93.92–95.31)
	CIN3+	93.23 (91.03–95.43)	10.53 (5.78–15.28)	96.48 (95.92–97.05)	10.53 (5.78–15.28)	96.48 (95.92–97.05)

Abbreviations: CIN, cervical intraepithelial neoplasia; CIN2+, CIN2 and worse; CIN3+, CIN3 and worse; PPV, positive predictive value; NPV, negative predictive value; CI, confidence interval.

*Cobas vs HC2 *p*<0.001; Cobas/HC2 vs HPV16 *p*<0.001.

^#^ Cobas vs HC2 *p* = 0.083 (CIN2+) and *p* = 0.157 (CIN3+); Cobas/HC2 vs HPV16 *p*<0.001.

^$^ Cobas vs HC2 *p*<0.001; Cobas/HC2 vs HPV16 *p*<0.001.

### Performance of HPV test in primary cervical screening

The effectiveness of co-testing versus cytology alone in primary cervical cancer screening was analyzed based on 6345 cases in the present comparative study (Table 6). Both the detection rates of CIN2+ and CIN3+ were significantly higher in the co-testing group compared to the cytology alone group (CIN2+: 0.53% vs 0.19%, OR 2.83, 1.17–7.84, *p* = 0.029; CIN3: 0.38% vs 0.06%, OR 5.98, 1.63–38.5, *p* = 0.019) in the baseline round screening. On the other hand, the CIN2+ detection was less in co-testing group than in cytology alone group in the second round (0.05% vs 0.19%, OR 0.26 0.01–1.17, *p* = 0.23). Over the two rounds of screening, the total detection of CIN2+/CIN3+ was still higher in co-testing group (CIN2+: 0.57% vs 0.32%, OR 1.79, 0.84–4.05; CIN3+: 0.41% vs 0.19%, OR 2.16, 0.85–6.16), though not reaching to statistical significance (*p* = 0.139 and *p* = 0.119).

**Table 6:** Comparison of CIN2+ and CIN3+ detection rates in co-testing and cytology alone groups.

All women (6345)		Co-testing N, % (95%CI)	Cytology alone N, % (95%CI)	OR* (95% CI)	*P* value
Baseline round	No. of women	3177	3168		
	CIN2+	17, 0.53% (0.33–0.86)	6, 0.19% (0.09–0.41)	2.83 (1.17–7.84)	0.029
	CIN3+	12, 0.38% (0.22–0.66)	2, 0.06% (0.02–0.23)	5.98 (1.63–38.5)	0.019
Subsequent round	No. of women	1982	2083		
	CIN2+	1, 0.05% (0.009–0.29)	4, 0.19% (0.07–0.49)	0.26 (0.01–1.78)	0.230
	CIN3+	1, 0.05% (0.009–0.29)	4, 0.19% (0.07–0.49)	0.26 (0.01–1.78)	0.230

Abbreviations: CIN, cervical intraepithelial neoplasia; CIN2+, CIN2 and worse; CIN3+, CIN3 and worse; OR, Odd ratio; CI, confidence interval.

*: co-testing vs cytology alone.

## Discussion

The present study was a sub-study of our previous RCT study with two rounds of screening to evaluate the effectiveness of co-testing and cytology alone in cervical cancer screening in a Chinese population cohort [17]. In previous RCT study, we demonstrated that the detection of CIN2+ or CIN3+ was significantly higher by co-testing compared to cytology alone in the first round (CIN2+: 0.95% vs 0.38%, OR 2.50, 95% CI 1.65–3.88, *p*<0.001; CIN3+: 0.62% vs 0.20%, OR 3.06, 95% CI 1.78–5.58, *p*<0.001), leading to the significantly reduction of the CIN2+/CIN3+ detection in subsequent round in the co-testing group (CIN2+: 0.08% vs 0.35%, OR 0.23, 95% CI 0.08–0.57, *p* = 0.003; CIN3+: 0.07% vs 0.24%, OR 0.27, 95% CI 0.08–0.76, *p* = 0.22). Over two rounds of screening, the cumulative CIN2+/CIN3+ detection was significantly higher in the co-testing groups compared to cytology alone group (CIN2+: 1.01% vs 0.66%, OR 1.53, 95% CI 1.09–2.19, *p* = 0.016; CIN3+: 0.67% vs 0.39%, OR 1.71, 95% CI 1.10–2.69, *p* = 0.018). Results of RCT study confirmed that the addition of a HR-HPV test to cytology for primary cervical cancer screening led to early detection of clinically significant preinvasive lesions, suggesting that co-testing may be more effective in reducing the risk of cervical cancer than cytology alone. Our result was in agreement with other studies showing that co-testing had a higher sensitivity than cytology alone [19–22]. The present study also demonstrated that adding HPV testing resulted in early detection of clinically significant precancerous lesions, despite of the smaller study cohort (only 6345 women from RCT study were assessed with both Cobas and HC2 tests) (Table 6).

As HPV testing is an increasing important part of cervical cancer screening, effective implementation of HPV test requires comprehensively validations of the test. Present study compared the analytical and clinical performance of Cobas test to HC2 test, a standard HPV test, in a Chinese population-based cohort. Overall, two tests were found comparable with 92.23% (95% CI: 91.57–92.89) agreement. There was no significant difference in identifying HR-HPV in cases associated with diseases between two tests, indicating that both tests were equally good in detecting cervical disease. In addition, the analysis of clinical performance showed that Cobas test had a significantly higher test specificity and better agreement with CIN2+/CIN3+ than HC2 test (Table 5), while the sensitivity of two tests for detection of high-grade CIN did not differ significantly (*p* = 0.083 and *p* = 0.157). Our results suggested that Cobas test demonstrated a comparable clinical performance to HC2 test in a screening. Similar findings were also observed in other studies [13, 15, 23–25]. It is important to know that in the previous RCT study women were managed by their HC2 and cytology results, whereas Cobas results were not revealed. Thus, women who detected with Cobas+/HC2-, were not referred to colposcopy unless they had abnormal cytology at baseline or subsequent round. Their histopathology data were not able to obtain. There were 135 Cobas+/HC2- cases found in co-testing group in this sub-study and none of them were referred to colposcopy for further assessment. It is possible that additional potential CIN lesions might have been identified among them, then would result in increased sensitivity for Cobas and decreased sensitivity for HC2 test.

We noticed that Cobas and HC2 tests only moderately agreed with each other (Ƙ = 0.493) in our study. There were some factors in the study would interfere the comparison analysis. First, our study was performed in a large population-based screening cohort and the majority of the smear samples were cytological negative (98.64%), which strongly influencing Ƙ-coefficient in statistical analysis [16, 26]. Secondly, it has been suggested that discordant results from comparison study may be more likely to be associated with low viral load [15, 26, 27]. In our study, a large difference in test signal strength, an indication of viral load, was observed between concordant positive samples and discordant samples (Table 2). Discordant results were frequently found in samples with Cobas Ct value and/or HC2 RLU/CO ratio closer to the test limits of the detection than concordant samples (Table 2). Hence, these discordant samples were most likely to contain lower viral loads, leading to the discrepant results from different tests. Lastly, we observed a correlation between the severity of underlying biology and the inter-assay agreement, with an increasing concordance found in cases with more severe abnormalities. There was no significant disagreement between two tests in CIN2+/CIN3+ samples. In Danish Horizon study, Rebolj and colleagues have reported that the percentage of HPV positive women testing positive on all four HPV assays increased from 22% in women with normal cytology to 68% in women with abnormal cytology and to 84% in women with ≥CIN2 [28]. The high level of concordance between HPV tests corresponded to a substantially higher likelihood of detecting high-grade lesions [13, 29, 30].

In addition, a higher positive rate of HR-HPV detection was observed in Cobas test than HC2 test (8.75% vs 7.97%) in present study. Our HPV positivity rates in the total study population were similar to the results of the recent Canadian FOCAL study [15], where HR-HPV positive rates of Cobas and HC2 were 8.8% and 8.4%, respectively. A higher positive HR-HPV rate in Cobas test compared with HC2 test was also reported by other studies [31, 32]. One explanation could be due to the insufficiency of the samples used for HC2 test. Unlike Cobas test, which used human β-globin gene as an internal control, HC2 test lacks internal control to monitor the specimen cellularity, so a negative result of HC2 test may be because of the sample inadequacy or test failure. Lacking internal control may introduce the potential for HC2 test to report false-negative results. Overall, majority of the concordant Cobas+/HC2+ cases (92.14%) were tested positive for HR-HPV by LA test. Approximately 25% (32/130) of Cobas-/HC2+ cases tested positive for LR-HPV by LA test, whereas only 7.5% (20/268) of Cobas+/HC2- cases did. This difference was statistically significant, indicating that HC2 test is more cross-reactivity with LR-HPV genotypes than Cobas test. The HC2 test has been reported to cross-react with some LR-HPV genotypes [15, 28, 32, 33] and the degree of the cross-reactivities were frequently more than that found in Cobas test. Some previous studies have also showed that the specificity of HC2 was compromised by cross-reactivity in its HR-HPV probe cocktail with certain untargeted, non-oncogenic HPV types [34–36]. Cross-reactivity with LR-HPV genotypes may result in false positives in women infected with only LR-HPV genotypes. Such infections do not cause malignant neoplasms and are clinically irrelevant. However, in a screening population, it is potentially harmful because they trigger unnecessary colposcopy referral and over-treatment. There was a 4.5-fold higher referral in co-testing group than cytology alone group in our previous RCT study [17] based on HC2 test results.

The added advantage of integrated genotyping of HPV16 and HPV18 in Cobas test has been demonstrated to be able to identify women at high risk of developing high-grade cervical lesions and cancer [14, 15, 37, 38]. However, the clinical value of HPV16 and HPV18 genotyping could not be recognized in the present study, albeit our overall HR-HPV positivity rates, including overall HR-HPV, HPV18 and 12 OHR were comparable with the recent Canadian FOCAL study (8.75% vs 8.8%, 0.61% vs 0.7%, 6.98% vs 7.1%) [15], except a lower HPV16 detection rate (1.15% vs 2.1%). HPV16 positive was identified in only 10.53% (2/19) CIN3 cases and HPV18 was negative in all CIN2+ cases. One of the reasons for these unexpected results may be due to a very low incidence rate of high-grade lesions (0.44%) detected in the study. This may be explained by the screening pattern in our screened cohort, where more than 90% of women had undergone yearly smear screening for many years prior to join the study. Therefore, our study population represented an over-screened population with a low prevalence of high-grade cervical lesion. Another reason may be owing to the study setting, where Cobas results were not used for patient management. Over 60% of HPV16+ (62.5%, 25/40) and HPV18+ (61.1%, 11/18) women in co-testing group were not referred to colposcopy. It is possible that additional CIN lesion would have been detected among these HPV16+/18+ women.

The strength of our study is its large sample size, which allowed a statistically powerful and detailed analysis of Cobas performance in comparison to HC2 test. However, our study was limited to a large convenient sample from women initially enrolled in an RCT study, using the leftover ThinPrep specimens collected after cytology and HC2 tests for Cobas and LA tests. As Cobas test was not used for patient management in previous RTC study, the missing histological data in discordant Cobas+/HC2- cases may have impact on the assessment of the sensitivity and specificity of the Cobas test. Another limitation of the present study was inherited from the previous RCT study, the study cohort was a highly screened population, leading to a low incidence of high-grade lesions. This may also constrain the present comparison study.

## Conclusions

Our large comparison study demonstrated that addition of a HR-HPV test to cytology for primary cervical screening may be more effective in reducing CIN2+ risk than cytology alone. The Cobas test demonstrated significantly higher specificity in identifying CIN2+/CIN3+ cases than HC2 test with comparable sensitivity of both tests in clinical evaluation. Due to the study limitations, the added benefit of HPV16 and HPV18 genotyping of Cobas test in clinical performance has not be acknowledged and need to be further evaluated in a screening cohort with increasing number of high-grade lesions.

## Supporting information

S1 Checklist CONSORT 2010 checklist of information to include when reporting a randomised trial. (PDF)

S1 Fig (PDF)

S1 Protocol (DOC)
