# Supplementary figures and images for: Clinical performance of the Roche Cobas 4800 HPV test for primary cervical cancer screening in a Chinese population
Source: PLoS One. 2022 Aug 5;17(8):e0272721. doi: 10.1371/journal.pone.0272721 (PMC9355206; doi:10.1371/journal.pone.0272721)

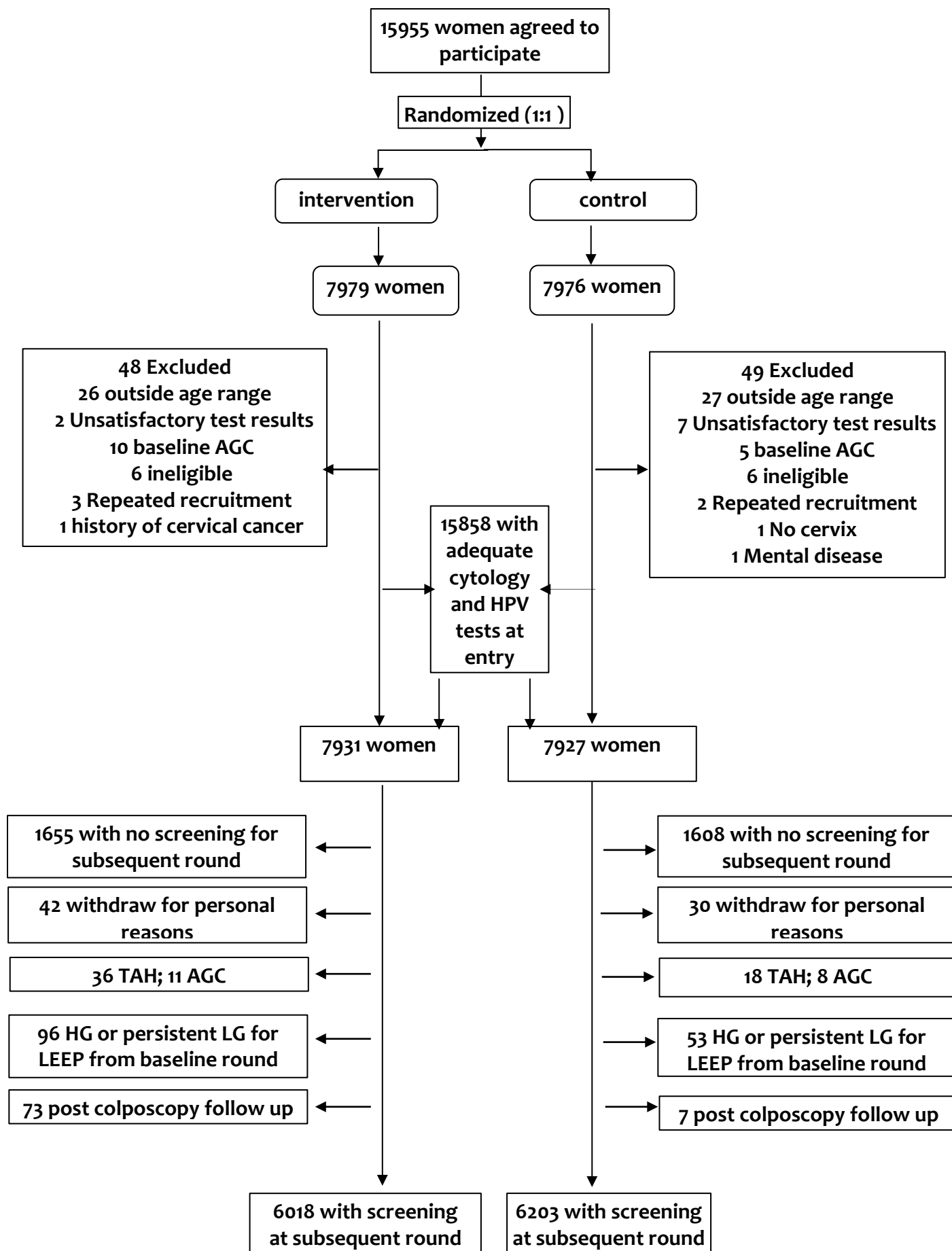

Supplement: S1 Fig — (PDF) [file pone.0272721.s002.pdf]
