# Supplementary material for: Clinical performance of the Roche Cobas 4800 HPV test for primary cervical cancer screening in a Chinese population
Source: PLoS One. 2022 Aug 5;17(8):e0272721. doi: 10.1371/journal.pone.0272721 (PMC9355206; doi:10.1371/journal.pone.0272721)
Supplement: S1 Protocol — (DOC) [file pone.0272721.s003.doc]

**“A randomized controlled trial comparing concomitant HPV–cytology testing with conventional cytology testing for the detection of high grade cervical intraepithelial neoplasia in primary cervical cancer screening in Hong Kong”**

**Research Protocol**

**INTRODUCTION**

With the introduction of cervical cancer screening since 1920s, the incidence and mortality rates of cervical cancer has decreased by 60-90%.[1] However, cervical cancer is still the second most common female cancer worldwide and it remains to be a global burden.[2] In Hong Kong, cervical cancer was the fifth commonest female cancer and there were 459 new cases and 133 mortality cases in 2006.[3] Conventional and liquid-based cytology is the traditional screening tool. However, its accuracy is limited by several factors such as the patients’ estrogen status, sampling techniques and variation in the interpretation of cytopathologists. The sensitivity and specificity of cytology testing were estimated to range from 30 – 90% and from 70 – 100% respectively.[4-9] Attempts have been made to look for new screening tools including human papillomavirus (HPV) testing, cervicography, visual inspection with acetic acid (VIA) and with Lugol’s iodine (VILI).

HPV is the causative agent of cervical cancer.[10-11] This strong relationship has attracted attention to evaluate the efficacy of applying HPV testing in the screening for cervical cancer and its pre-invasive lesions. Currently, HPV testing is commonly used in triaging patients with atypical squamous cells of undetermined significance (ASCUS) for colposcopy and biopsy.[12-15] Recent research has focused on the comparison of HPV testing with conventional cytology testing in primary screening. Despite the heterogeneity of the study designs and follow-up methods, randomized controlled trials and meta-analyses have shown that the sensitivity of HPV testing for the detection of cervical intraepithelial neoplasia (CIN) grade 2 or higher is above 90% in general, being 25 - 30% higher than that of cytology testing with a relative sensitivity ranging between 1.16 and 3.50.[16-27] Moreover, the implementation of HPV testing led to earlier detection of CIN 2 or worse and might potentially lengthen the screening interval to up to 5 or 6 years.[23, 28] Nevertheless, the specificity of HPV DNA test was 67 - 94%, being about 6% less than that of cervical cytology. The positive predictive value (PPV) was also lower, with the relative PPV being 0.58 – 0.99 depending on the age-group and the cutoff value of the viral load used.[23, 28]

On the other hand, one study demonstrated that the use of HPV and cytology testings in primary screening increased the sensitivity of detecting CIN 3 or cancer by 35% (95% confidence interval [CI] 15 – 60%) with an insignificant decrease of PPV (relative PPV 0.76, 95% CI 0.52 – 1.10).[27] Meanwhile, other similar studies detected a substantial reduction in the PPV (relative PPV 0.55, 95% CI 0.37 – 0.82 for women aged 25 – 34; relative PPV 0.40, 95% CI 0.23 – 0.66 for women aged 35 - 60).[29, 30] A recent cohort study in Taiwan showed that the addition of HPV testing to cytology testing improved the sensitivity by 15.3% with a decrease of specificity by 9.6%, while the addition of conventional smear to primary high-risk HPV testing improved the sensitivity by 12.5% with a decrease of specificity by 0.5%.[31] The controversy of these studies makes it difficult to determine whether co-testing is cost-effective, and the complexity of different screening and follow-up strategies also fail to identify the best approach. In addition, there is no data on the performance of cytology and HPV testing in the local Chinese population. Therefore, our objective is to conduct a prospective study to compare the use of concomitant HPV-cytology testing with conventional cytology testing in primary cervical screening for the detection of high grade CIN among Hong Kong Chinese women.

**METHODOLOGY**

**Study Aim**

To compare HPV-cytology co-testing with cytology testing in primary cervical screening for the detection of high grade cervical intraepithelial neoplasia (CIN2 or higher) among Hong Kong Chinese women aged 30 years or above.

**Subjects**

*Inclusion criteria*

- Ethnic Chinese women aged 30 to 60 years who have completed a written consent
- Women who have not attended screening for the past 3 years or more will be given priority

*Exclusion criteria*

- Currently pregnant
- Without a cervix
- Congenital abnormalities of the lower genital tract
- Previous history of invasive cervical cancer
- Who has been followed-up or treated for an abnormal cytology result in the past 12 months
- Who are unable to provide consent

*Recruitment*

Women attending the women’s health clinics of the Family Planning Association of Hong Kong or the Lady Helen Woo Women’s Diagnostic and Treatment Centre for routine cervical cancer screening will be recruited. Eligible women will be given an information sheet containing details on cervical cancer and HPV testing and about the present study.(App. A & B) Women who are interested to participate will be asked to complete an informed consent (App. A & B) and a self-administered questionnaire (App. C & D) to collect baseline data on personal demographics, past screening history, behavioural risk factors for cervical cancer and other information related to ancillary cost-effectiveness study and psychosocial studies.

**Study Design**

This is a prospective randomized controlled trial to study the effect of HPV-cytology co-testing as a primary screening strategy for women aged 30 years or above. Each consented participant will be randomized on a 1:1 ratio into one of two arms: Cytology arm (control) or cytology plus HPV testing arm (intervention). Participants will be followed for two rounds of screening or until the occurrence of high grade CIN, whichever takes place first. Cytology testing, HPV testing and histological studies of biopsies will be conducted by the laboratories of the University of Hong Kong. Colposcopy and biopsy, when indicated, will be performed by the colposcopists at the colposcopy clinic in Queen Mary Hospital or at the Lady Helen Woo Women’s Diagnostic and Treatment Centre. All final test reports will be reviewed and signed by the senior pathologists.

***Control arm***

The index test for participants in the control arm will be conventional cytology testing. HPV testing will also be administered. ,HPV results will not be revealed to the participant or healthcare professionals who are involved in the direct care of the participant except in the case of reflex HPV testing for borderline cytology results. Participants will be managed based on their cytology results according to the current practice of the involved clinics and the Hong Kong College of Obstetricians and Gynaecologists Guidelines. (Fig. 1) Colposcopy will be offered in the following conditions: (1) any cytologic abnormalities greater than ASCUS, (2) ASCUS and HPV positive, or (3) two consecutive ASCUS results. In addition, a random 5% of participants with negative cytology results will be invited to attend colposcopy at baseline at the Lady Helen Woo Women’s Diagnostic and Treatment Centre.  Post colposcopy management of participants is shown in Fig. 2. Participation in the study will be terminated at a diagnosis of CIN 2 or greater.

***Intervention arm***

The index test in the intervention arm will be concomitant cytology testing and HPV testing. Participants will be managed based on both their cytology and HPV results in accordance with the routine practice of the involved clinics, the Hong Kong College of Obstetricians and Gynaecologists Guidelines and expert opinion on the management following a positive HPV testing result. (Fig. 3) Participants who are HPV negative with borderline cytology at baseline will be re-tested in 12 months by cytology and HPV testings. For this study, HPV positivity is defined as the presence of one or more high-risk HPV subtypes. Colposcopy will be offered in the following conditions: (1) any cytologic abnormalities greater than ASCUS regardless of the HPV result or (2) two consecutive ASCUS results regardless of the HPV results or (3) HPV positivity. Post colposcopy management of participants is shown in Fig. 4. Participation in the study will be terminated at a diagnosis of CIN 2 or greater.

***Randomization***

A computer-generated block randomization algorithm with randomly varied block size will be used to randomize participants 1:1 into one of the two arms. Randomization will be carried out away from the recruitment sites at the central study base at the Department of O&G of the University of Hong Kong. Each cervical sample will be assigned a study ID at the recruitment site. Arm allocation information corresponding to the study ID of individual sample will be revealed to the processing laboratory.

***Blinding***

Participants and recruitment staff will be blinded to the arm allocation at recruitment. Participants will be informed of their cytology and/or HPV results if necessary for subsequent follow-up. Cytology testing and HPV testing will be conducted by two separate teams of laboratory staff and they will be blinded to each other’s test results. Colposcopists will be blinded to the cytology and HPV results.

***Sample Collection***

At the recruitment clinics, the liquid based cervical cytology samples will be collected by Papette^TM^ collection devices, a broom-type sampling device and then rinsed directly into the PreservCyt vial. Women attending these clinics can be considered to be representing the Hong Kong screening population since the prevalence of low-grade and more severe SIL was less than 5%.[32, 34, 35]

***Cytology testing***

The labeled samples will be sent to the Cervical Cytology Laboratory, the University of Hong Kong and processed using TP 2000 Processors.[32] The specimen adequacy and the cytological features in these slides were then evaluated by the cytotechnologists using the Bethesda System.[36] Cytologic abnormalities including atypical squamous cells of undetermined significance (ASC-US) [33], atypical squamous cells cannot exclude a high-grade lesion (ASC-H), atypical glandular cells of undetermined significance (AGUS), low-grade squamous intraepithelial lesion (LSIL), high-grade squamous intraepithelial lesion (HSIL), and cancer were reported by pathologists.

***HPV testing***

Reflex HPV DNA testing will be performed by the laboratory of the Department of Pathology in case of a borderline cytology result at baseline for the control group. Other HPV DNA testing and HPV genotyping will be performed by the laboratory of the Department of O&G.

***Colposcopy and biopsy***

Colposcopy is performed using the Olympus Optical System. A speculum would be inserted into the vagina to expose the cervix. The cervix will be cleaned with normal saline and inspected under the colposcope with different magnifications. The cervix will then be applied with 3% acetic acid and be inspected with the colposcope in different magnifications. Acetowhite areas as well as abnormal vascular patterns will be noted during the examination. Lugol’s Iodine will be applied as an adjunct for the colposcopy examination. Cervical biopsies will be taken from the most suspicious areas in each quadrant of the cervix. If no abnormality was found at a particular quadrant, a random biopsy will be taken so that, at least one cervical biopsy will be taken from each quadrant. Endocervical curettage would be performed in all the cases. After the examination of the cervix, the vagina will be inspected. Vaginal biopsy will be taken only if lesions are identified. Haemostasis after cervical biopsy will be achieved using the Monsel’s solution. Additional methods like Silver Nitrate sticks or vaginal packing with the gauze roll will be used when necessary.

***Cervical Biopsies Reporting***

The cervical biopsy specimens and the biopsy request will be sent to the Cervical Screening Laboratory. After specimen identification and labeling, the specimens and forms with accession number will be transferred to the Histopathology Laboratory, the University of Hong Kong. The tissue will undergo procedures to produce thin sections for microscopic examinations, inlcuding tissue processing, embedding, microtome sectioning, and Haematoxylin and Eosin staining.

The pathologists on duty may be requested to perform macroscopic description and cut up for bigger specimens such as loop excision. After tissue processing, histology slides and the forms are then returned to the office of Cervical Screening Laboratory for distribution to pathologists on duty. The macroscopic and microscopic descriptions as well as diagnosis are entered into the same laboratory information system.

**Outcome Measures**

Histological CIN2, CIN3 and cervical cancer are the primary outcomes of the study. Diagnosis will be obtained through biopsies. Secondary outcomes include (1) HPV prevalence at baseline, (2) HPV clearance or persistence among participants who test positive at baseline, (3) clearance of mild cervical abnormalities among participants who test HPV negative and (4) sensitivities, specificities, predictive values of the two screening methods.

**Hypotheses**

- There is a significant difference in the number of CIN2+ cases detected between the cytology testing group and the cytology-HPV co-testing group at baseline.
- Significantly more CIN2+ cases will be detected at the second round of screening among participants with normal cytology result in the control arm than those with normal cytology and negative HPV results in the intervention arm.
- Cases infected by certain HPV genotypes are at higher risk of developing CIN2+ or HGSIL than those infected by other HPV genotypes.

**Sample Size Calculation**

Based on the 2004-07 registry data from the Department of Health’s Cervical Screening Programme, the local prevalence of CIN2+ was postulated to be about 1.2%. A total sample size of 12000 women will be required to detect an absolute difference of 25% in the sensitivity between the control arm and the intervention arm in the detection of CIN2+ at 80% power and alpha = 0.05 (2-sided) and to account for an estimated 10% lost to follow-up rate.

**Data analysis**

Data analysis will be performed according to the intention-to-treat principle. For proportion comparisons, chi-square tests will be employed. At baseline, logistic regression will be used to determine the associations between cervical cancer risk factors and HPV infection or cervical abnormalities. HPV prevalence will be calculated for participants in the intervention group. HPV clearance or persistence will be obtained by comparing those participants who have a positive HPV result at baseline and who do not require treatment for cervical lesions with their subsequent HPV testing results at 1-year post colposcopy. Clearance of mild cervical abnormalities will be obtained based on the participants with borderline cytology results at baseline and the proportion of these same participants who turn normal at subsequent screening without ever requiring treatment for cervical precancerous lesions if present. Incidence of CIN2+ will be calculated for both arms at baseline and at the second screening round. The estimated relative risks of CIN2+ will be calculated and compared at the second screening round for the control arm participants who have normal cytology results and for those in the intervention arm who have normal cytology and negative HPV results at baseline, respectively. Sensitivities, specificities, predictive values of the two screening methods for the detection of CIN2+ will be calculated based on the cervical biopsy data from participants who undergo colposcopy at baseline.

**Duration of study**

Subject recruitment will take two years and a minimum of five years will be required to complete the second round of screening for all participants. Another year will be required for the completion of all reports, data entry and cleaning, statistical analysis and writing up of final report. The whole duration of the study is estimated to last from eight to ten years.

**REFERENCES**

1. Anttila A, Läärä E. In: Sankila R, Démaret E, Hakama M, Lynge E, Schouten LJ, Parkin DM, for the European Network of Cancer Registries. Evaluating and monitoring of screening programmes. Brussels/Luxembourg: European Commission, Europe Against Cancer Programme, 2000.
2. Parkin DM, Bray F, Ferlay J, Pisani P. [Global cancer statistics, 2002.](http://www.ncbi.nlm.nih.gov/pubmed/15761078?ordinalpos=4&itool=EntrezSystem2.PEntrez.Pubmed.Pubmed_ResultsPanel.Pubmed_DefaultReportPanel.Pubmed_RVDocSum) CA Cancer J Clin 2005; 55: 74 - 108.
3. Hong Kong Cancer Stat 2006. Hong Kong Cancer Registry. Hong Kong: Hospital Authority; 2008 (http://www3.ha.org.hk/cancereg)
4. Nanda K, McCrory DC, Myers ER, Bastian LA, Hasselblad V, Hickey JD, Matchar DB. [Accuracy of the Papanicolaou test in screening for and follow-up of cervical cytologic abnormalities: a systematic review.](http://www.ncbi.nlm.nih.gov/pubmed/10819705?ordinalpos=6&itool=EntrezSystem2.PEntrez.Pubmed.Pubmed_ResultsPanel.Pubmed_DefaultReportPanel.Pubmed_RVDocSum) Ann Intern Med 2000; 132: 810 - 9.
5. Coste J, Cochand-Priollet B, de Cremoux P, Le Galès C, Cartier I, Molinié V, Labbé S, Vacher-Lavenu MC, Vielh P; French Society of Clinical Cytology Study Group. [Cross sectional study of conventional cervical smear, monolayer cytology, and human papillomavirus DNA testing for cervical cancer screening.](http://www.ncbi.nlm.nih.gov/pubmed/12676841?ordinalpos=5&itool=EntrezSystem2.PEntrez.Pubmed.Pubmed_ResultsPanel.Pubmed_DefaultReportPanel.Pubmed_RVDocSum) BMJ 2003; 326: 733.
6. Strander B, Andersson-Ellström A, Milsom I, Rådberg T, Ryd W. [Liquid-based cytology versus conventional Papanicolaou smear in an organized screening program: a prospective randomized study.](http://www.ncbi.nlm.nih.gov/pubmed/17724676?ordinalpos=4&itool=EntrezSystem2.PEntrez.Pubmed.Pubmed_ResultsPanel.Pubmed_DefaultReportPanel.Pubmed_RVDocSum) Cancer 2007; 111: 285 - 91.
7. Arbyn M, Bergeron C, Klinkhamer P, Martin-Hirsch P, Siebers AG, Bulten J. [Liquid compared with conventional cervical cytology: a systematic review and meta-analysis.](http://www.ncbi.nlm.nih.gov/pubmed/18165406?ordinalpos=3&itool=EntrezSystem2.PEntrez.Pubmed.Pubmed_ResultsPanel.Pubmed_DefaultReportPanel.Pubmed_RVDocSum) Obstet Gynecol 2008; 111: 167 - 77.
8. Sykes PH, Harker DY, Miller A, Whitehead M, Neal H, Wells JE, Peddie D. [A randomised comparison of SurePath liquid-based cytology and conventional smear cytology in a colposcopy clinic setting.](http://www.ncbi.nlm.nih.gov/pubmed/18823488?ordinalpos=2&itool=EntrezSystem2.PEntrez.Pubmed.Pubmed_ResultsPanel.Pubmed_DefaultReportPanel.Pubmed_RVDocSum) BJOG 2008; 115: 1375 - 81.
9. Beerman H, van Dorst EB, Kuenen-Boumeester V, Hogendoorn PC. [Superior performance of liquid-based versus conventional cytology in a population-based cervical cancer screening program.](http://www.ncbi.nlm.nih.gov/pubmed/19150573?ordinalpos=1&itool=EntrezSystem2.PEntrez.Pubmed.Pubmed_ResultsPanel.Pubmed_DefaultReportPanel.Pubmed_RVDocSum) Gynecol Oncol 2009; 112: 572 - 6.
10. Bosch FX, Manos MM, Muñoz N, Sherman M, Jansen AM, Peto J, Schiffman MH, Moreno V, Kurman R, Shah KV. [Prevalence of human papillomavirus in cervical cancer: a worldwide perspective. International biological study on cervical cancer (IBSCC) Study Group.](http://www.ncbi.nlm.nih.gov/pubmed/7791229?ordinalpos=96&itool=EntrezSystem2.PEntrez.Pubmed.Pubmed_ResultsPanel.Pubmed_DefaultReportPanel.Pubmed_RVDocSum) J Natl Cancer Inst 1995; 87: 796 - 802.
11. Walboomers JM, Jacobs MV, Manos MM, Bosch FX, Kummer JA, Shah KV, Snijders PJ, Peto J, Meijer CJ, Muñoz N. [Human papillomavirus is a necessary cause of invasive cervical cancer worldwide.](http://www.ncbi.nlm.nih.gov/pubmed/10451482?ordinalpos=21&itool=EntrezSystem2.PEntrez.Pubmed.Pubmed_ResultsPanel.Pubmed_DefaultReportPanel.Pubmed_RVDocSum) J Pathol 1999; 189: 12 - 9.
12. Solomon D, Schiffman M, Tarone R; ALTS Study group. [Comparison of three management strategies for patients with atypical squamous cells of undetermined significance: baseline results from a randomized trial.](http://www.ncbi.nlm.nih.gov/pubmed/11181776?ordinalpos=1&itool=EntrezSystem2.PEntrez.Pubmed.Pubmed_ResultsPanel.Pubmed_DefaultReportPanel.Pubmed_RVDocSum) J Natl Cancer Inst 2001; 93: 293 - 9.
13. ASCUS-LSIL Traige Study (ALTS) Group. [Results of a randomized trial on the management of cytology interpretations of atypical squamous cells of undetermined significance.](http://www.ncbi.nlm.nih.gov/pubmed/12824967?ordinalpos=2&itool=EntrezSystem2.PEntrez.Pubmed.Pubmed_ResultsPanel.Pubmed_DefaultReportPanel.Pubmed_RVDocSum) Am J Obstet Gynecol 2003; 188: 1383 - 92.
14. Kulasingam SL, Kim JJ, Lawrence WF, Mandelblatt JS, Myers ER, Schiffman M, Solomon D, Goldie SJ; ALTS Group. [Cost-effectiveness analysis based on the atypical squamous cells of undetermined significance/low-grade squamous intraepithelial lesion Triage Study (ALTS).](http://www.ncbi.nlm.nih.gov/pubmed/16418511?ordinalpos=3&itool=EntrezSystem2.PEntrez.Pubmed.Pubmed_ResultsPanel.Pubmed_DefaultReportPanel.Pubmed_RVDocSum) J Natl Cancer Inst 2006; 98: 92 - 100.
15. Arbyn M, Paraskevaidis E, Martin-Hirsch P, Prendiville W, Dillner J. [Clinical utility of HPV-DNA detection: triage of minor cervical lesions, follow-up of women treated for high-grade CIN: an update of pooled evidence.](http://www.ncbi.nlm.nih.gov/pubmed/16154623?ordinalpos=19&itool=EntrezSystem2.PEntrez.Pubmed.Pubmed_ResultsPanel.Pubmed_DefaultReportPanel.Pubmed_RVDocSum) Gynecol Oncol 2005; 99 (3 Suppl 1): S7 - 11.
16. Arbyn M, Buntinx F, Van Ranst M, Paraskevaidis E, Martin-Hirsch P, Dillner J. [Virologic versus cytologic triage of women with equivocal Pap smears: a meta-analysis of the accuracy to detect high-grade intraepithelial neoplasia.](http://www.ncbi.nlm.nih.gov/pubmed/14970277?ordinalpos=11&itool=EntrezSystem2.PEntrez.Pubmed.Pubmed_ResultsPanel.Pubmed_DefaultReportPanel.Pubmed_RVDocSum) J Natl Cancer Inst 2004; 96: 280 - 93.
17. Kotaniemi-Talonen L, Nieminen P, Anttila A, Hakama M. [Routine cervical screening with primary HPV testing and cytology triage protocol in a randomised setting.](http://www.ncbi.nlm.nih.gov/pubmed/16189520?ordinalpos=10&itool=EntrezSystem2.PEntrez.Pubmed.Pubmed_ResultsPanel.Pubmed_DefaultReportPanel.Pubmed_RVDocSum) Br J Cancer 2005; 93: 862 - 7.
18. Arbyn M, Sasieni P, Meijer CJ, Clavel C, Koliopoulos G, Dillner J. [Chapter 9: Clinical applications of HPV testing: a summary of meta-analyses.](http://www.ncbi.nlm.nih.gov/pubmed/16950021?ordinalpos=9&itool=EntrezSystem2.PEntrez.Pubmed.Pubmed_ResultsPanel.Pubmed_DefaultReportPanel.Pubmed_RVDocSum) Vaccine 2006; 24 Suppl 3: S3 / 78 - 89.
19. Cuzick J, Clavel C, Petry KU, Meijer CJ, Hoyer H, Ratnam S, Szarewski A, Birembaut P, Kulasingam S, Sasieni P, Iftner T. [Overview of the European and North American studies on HPV testing in primary cervical cancer screening.](http://www.ncbi.nlm.nih.gov/pubmed/16586444?ordinalpos=8&itool=EntrezSystem2.PEntrez.Pubmed.Pubmed_ResultsPanel.Pubmed_DefaultReportPanel.Pubmed_RVDocSum) Int J Cancer 2006; 119: 1095 - 101.
20. Koliopoulos G, Arbyn M, Martin-Hirsch P, Kyrgiou M, Prendiville W, Paraskevaidis E. [Diagnostic accuracy of human papillomavirus testing in primary cervical screening: a systematic review and meta-analysis of non-randomized studies.](http://www.ncbi.nlm.nih.gov/pubmed/17084886?ordinalpos=7&itool=EntrezSystem2.PEntrez.Pubmed.Pubmed_ResultsPanel.Pubmed_DefaultReportPanel.Pubmed_RVDocSum) Gynecol Oncol 2007; 104: 232 - 46.
21. Ronco G, Brezzi S, Carozzi F, Dalla Palma P, Giorgi-Rossi P, Minucci D, Naldoni C, Segnan N, Zappa M, Zorzi M, Cuzick J; NTCC study group. [The New Technologies for Cervical Cancer Screening randomised controlled trial. An overview of results during the first phase of recruitment.](http://www.ncbi.nlm.nih.gov/pubmed/17822751?ordinalpos=6&itool=EntrezSystem2.PEntrez.Pubmed.Pubmed_ResultsPanel.Pubmed_DefaultReportPanel.Pubmed_RVDocSum) Gynecol Oncol 2007; 107 (1 Suppl 1): S230 - 2.
22. Mayrand MH, Duarte-Franco E, Rodrigues I, Walter SD, Hanley J, Ferenczy A, Ratnam S, Coutlée F, Franco EL; Canadian Cervical Cancer Screening Trial Study Group. [Human papillomavirus DNA versus Papanicolaou screening tests for cervical cancer.](http://www.ncbi.nlm.nih.gov/pubmed/17942871?ordinalpos=1&itool=EntrezSystem2.PEntrez.Pubmed.Pubmed_ResultsPanel.Pubmed_DefaultReportPanel.Pubmed_RVDocSum) N Engl J Med 2007; 357: 1579 - 88.
23. Bulkmans NW, Berkhof J, Rozendaal L, van Kemenade FJ, Boeke AJ, Bulk S, Voorhorst FJ, Verheijen RH, van Groningen K, Boon ME, Ruitinga W, van Ballegooijen M, Snijders PJ, Meijer CJ. [Human papillomavirus DNA testing for the detection of cervical intraepithelial neoplasia grade 3 and cancer: 5-year follow-up of a randomised controlled implementation trial.](http://www.ncbi.nlm.nih.gov/pubmed/17919718?ordinalpos=5&itool=EntrezSystem2.PEntrez.Pubmed.Pubmed_ResultsPanel.Pubmed_DefaultReportPanel.Pubmed_RVDocSum) Lancet 2007; 370: 1764 - 72.
24. Kotaniemi-Talonen L, Anttila A, Malila N, Tarkkanen J, Laurila P, Hakama M, Nieminen P. [Screening with a primary human papillomavirus test does not increase detection of cervical cancer and intraepithelial neoplasia 3.](http://www.ncbi.nlm.nih.gov/pubmed/18248809?ordinalpos=4&itool=EntrezSystem2.PEntrez.Pubmed.Pubmed_ResultsPanel.Pubmed_DefaultReportPanel.Pubmed_RVDocSum) Eur J Cancer 2008; 44: 565 - 71.
25. Ronco G, Giorgi-Rossi P, Carozzi F, Confortini M, Dalla Palma P, Del Mistro A, Gillio-Tos A, Minucci D, Naldoni C, Rizzolo R, Schincaglia P, Volante R, Zappa M, Zorzi M, Cuzick J, Segnan N; New Technologies for Cervical Cancer Screening Working Group. [Results at recruitment from a randomized controlled trial comparing human papillomavirus testing alone with conventional cytology as the primary cervical cancer screening test.](http://www.ncbi.nlm.nih.gov/pubmed/18364502?ordinalpos=3&itool=EntrezSystem2.PEntrez.Pubmed.Pubmed_ResultsPanel.Pubmed_DefaultReportPanel.Pubmed_RVDocSum) J Natl Cancer Inst 2008; 100: 492 - 501.
26. Cuzick J, Arbyn M, Sankaranarayanan R, Tsu V, Ronco G, Mayrand MH, Dillner J, Meijer CJ. [Overview of human papillomavirus-based and other novel options for cervical cancer screening in developed and developing countries.](http://www.ncbi.nlm.nih.gov/pubmed/18847555?ordinalpos=2&itool=EntrezSystem2.PEntrez.Pubmed.Pubmed_ResultsPanel.Pubmed_DefaultReportPanel.Pubmed_RVDocSum) Vaccine 2008; 26 Suppl 10: K29 - 41.
27. Naucler P, Ryd W, Törnberg S, Strand A, Wadell G, Elfgren K, Rådberg T, Strander B, Forslund O, Hansson BG, Hagmar B, Johansson B, Rylander E, Dillner J. [Efficacy of HPV DNA testing with cytology triage and/or repeat HPV DNA testing in primary cervical cancer screening.](http://www.ncbi.nlm.nih.gov/pubmed/19141778?ordinalpos=1&itool=EntrezSystem2.PEntrez.Pubmed.Pubmed_ResultsPanel.Pubmed_DefaultReportPanel.Pubmed_RVDocSum) J Natl Cancer Inst 2009; 101: 88 - 99.
28. Naucler P, Ryd W, Törnberg S, Strand A, Wadell G, Elfgren K, Rådberg T, Strander B, Johansson B, Forslund O, Hansson BG, Rylander E, Dillner J. [Human papillomavirus and Papanicolaou tests to screen for cervical cancer.](http://www.ncbi.nlm.nih.gov/pubmed/17942872?ordinalpos=10&itool=EntrezSystem2.PEntrez.Pubmed.Pubmed_ResultsPanel.Pubmed_RVDocSum) N Engl J Med 2007; 357: 1589 - 97.
29. Ronco G, Giorgi-Rossi P, Carozzi F, Dalla Palma P, Del Mistro A, De Marco L, De Lillo M, Naldoni C, Pierotti P, Rizzolo R, Segnan N, Schincaglia P, Zorzi M, Confortini M, Cuzick J; New Technologies for Cervical Cancer screening Working Group. [Human papillomavirus testing and liquid-based cytology in primary screening of women younger than 35 years: results at recruitment for a randomised controlled trial.](http://www.ncbi.nlm.nih.gov/pubmed/16814206?ordinalpos=16&itool=EntrezSystem2.PEntrez.Pubmed.Pubmed_ResultsPanel.Pubmed_RVDocSum) Lancet Oncol 2006; 7: 547 - 55.
30. Ronco G, Segnan N, Giorgi-Rossi P, Zappa M, Casadei GP, Carozzi F, Dalla Palma P, Del Mistro A, Folicaldi S, Gillio-Tos A, Nardo G, Naldoni C, Schincaglia P, Zorzi M, Confortini M, Cuzick J; New Technologies for Cervical Cancer Working Group. [Human papillomavirus testing and liquid-based cytology: results at recruitment from the new technologies for cervical cancer randomized controlled trial.](http://www.ncbi.nlm.nih.gov/pubmed/16757701?ordinalpos=17&itool=EntrezSystem2.PEntrez.Pubmed.Pubmed_ResultsPanel.Pubmed_RVDocSum) J Natl Cancer Inst 2006; 98: 765 - 74.
31. Chao A, Hsu KH, Lai CH, Huang HJ, Hsueh S, Lin SR, Jung SM, Chao FY, Huang SL, Huang CC, Yang JE, Chang TC. [Cervical cancer screening program integrating Pap smear and HPV DNA testing: a population-based study.](http://www.ncbi.nlm.nih.gov/pubmed/18338752?ordinalpos=1&itool=EntrezSystem2.PEntrez.Pubmed.Pubmed_ResultsPanel.Pubmed_DefaultReportPanel.Pubmed_RVDocSum) Int J Cancer 2008; 122: 2835 - 41.
32. Cheung AN, Szeto EF, Leung BS, Khoo US, Ng AW. Liquid-based cytology and conventional cervical smears: a comparison study in an Asian screening population. Cancer. 2003 Dec 25;99(6):331-5.
33. Cheung AN, Szeto EF, Ng KM, Fong KW, Yeung AC, Tsun OK, Khoo US, Chan KY, Ng AW. Atypical squamous cells of undetermined significance on cervical smears: follow-up study of an Asian screening population.Cancer. 2004 Apr 25;102(2):74-80.
34. Lee KR, Ashfaq R, Birdsong GG, Corkill ME, McIntosh KM, Inhorn SL. Comparison of conventional Papanicolaou smears and a fluid-based, thin-layer system for cervical cancer screening. *Obstet Gynecol* 1997; **90**: 278-84.
35. Jones W. Impact of the Bethesda System. *Cancer.* 1995; 76:1914-1918.
36. Solomon D, Davey D, Kurman R, et al. The 2001 Bethesda System. Terminology and reporting results of cervical cytology. *JAMA* 2002; 287:2114–2149.

**Fig. 1 Control arm (conventional cytology testing & reflex** HPV testing) n = 6000

|  | **1** |  |  |  |  | **2** |  |  |  | **3** |  |  |  |  | Rules:   1. Any > A 🡪 Bx 2. A & HPV+ 🡪 Bx 3. 5% (n = 277) of baseline C- 🡪 Bx*   4. A x 2 🡪 Bx  C = cytology  A = Ascus  Bx = colposcopy +/- biopsy |
| --- | --- | --- | --- | --- | --- | --- | --- | --- | --- | --- | --- | --- | --- | --- | --- |
| **0** | **C-***  **n = 5544** |  |  |  |  | **A**  **n = 261** |  |  |  | **>A**  **n = 145** |  |  |  |  |  |
|  |  |  |  |  |  |  |  |  |  | **Bx** |  |  |  |  |  |
|  |  |  |  |  |  | **reflex HPV testing** |  |  |  |  |  |  |  |  |  |
|  |  |  |  |  |  |  |  |  |  |  |  |  |  |  |  |
|  |  |  |  |  | **H-** |  | **H+** |  |  |  |  |  |  |  |  |
|  |  |  |  |  |  |  |  |  |  |  |  |  |  |  |  |
|  |  |  |  |  |  |  | **Bx** |  |  |  |  |  |  |  |  |
| **1y** |  |  |  |  | **Pap** |  |  |  |  |  |  |  |  |  |  |
|  |  |  |  |  |  |  |  |  |  |  |  |  |  |  |  |
|  |  |  |  | **C-** |  | **≧A** |  |  |  |  |  |  |  |  |  |
|  |  |  |  |  |  |  |  |  |  |  |  |  |  |  |  |
|  |  |  |  |  |  | **Bx** |  |  |  |  |  |  |  |  |  |
| **3y** | **Pap** |  |  |  |  |  |  |  |  |  |  |  |  |  |  |
|  |  |  |  |  |  |  |  |  |  |  |  |  |  |  |  |
| **4y** |  |  |  | **Pap** |  |  |  |  |  |  |  |  |  |  |  |
|  |  |  |  |  |  |  |  |  |  |  |  |  |  |  |  |

Fig. 2 Control arm (post colposcopy – biopsy)

| **0** |  |  |  |  |  |  |  |  |  |  | **Bx** |  | Rules:   1. After Bx, Pap x 3 q6m. If 3 consecutive C- 🡪 routine screening 2. After Bx, if 2 consecutive A/LSIL 🡪 repeat Bx 3. Any > CIN1 🡪 out study   *CIN 1 in > 2 quad or persist for > 2yrs 🡪 Tx  Bx = colposcopy +/- biopsy Tx = treatment  C = cytology  A = Ascus  PapR = routine screening |
| --- | --- | --- | --- | --- | --- | --- | --- | --- | --- | --- | --- | --- | --- |
|  |  |  |  |  |  |  |  | **Normal / HPV / CIN 1*** | |  |  | **CIN 2-3 / invasive** | |
|  |  |  |  |  |  |  |  |  |  |  |  | **Tx, Out** |  |
| **6m** | **post** |  |  |  |  |  | **Pap** |  |  |  |  |  |  |
|  |  |  |  |  |  |  |  |  |  |  |  |  |  |
|  |  |  |  |  |  |  |  |  |  |  |  |  |  |
|  |  |  |  |  | **C- / A / LSIL** |  |  |  | **HSIL** |  |  |  |  |
|  |  |  |  |  |  |  |  |  |  |  |  |  |  |
|  |  |  |  |  |  |  |  |  | **Bx** |  |  |  |  |
| **1y** | **post** |  |  |  | **Pap** |  |  |  |  |  |  |  |  |
|  |  |  |  |  |  |  |  |  |  |  |  |  |  |
|  |  |  |  |  |  |  |  |  |  |  |  |  |  |
|  |  |  | **C-** |  | **≧A** |  |  |  |  |  |  |  |  |
|  |  |  |  |  |  |  |  |  |  |  |  |  |  |
|  |  |  |  |  | **Bx** |  |  |  |  |  |  |  |  |
|  |  |  |  |  |  |  |  |  |  |  |  |  |  |
| **1.5 y** | **post** |  | **Pap** |  |  |  |  |  |  |  |  |  |  |
|  |  |  |  |  |  |  |  |  |  |  |  |  |  |
|  |  |  | **C-** | **≧A** |  |  |  |  |  |  |  |  |  |
|  |  |  |  |  |  |  |  |  |  |  |  |  |  |
| **4.5 y** | **post** |  | **PapR** |  |  |  |  |  |  |  |  |  |  |
|  |  |  |  |  |  |  |  |  |  |  |  |  |  |

Fig. 3 Intervention arm (conventional cytology testing & HPV testing) n = 6000

|  | **1** |  |  |  | **2** |  |  |  |  | **3** |  |  |  | **4** |  |  | **5** |
| --- | --- | --- | --- | --- | --- | --- | --- | --- | --- | --- | --- | --- | --- | --- | --- | --- | --- |
| **0** | **H-,C-** |  |  |  | **H-,A**  **n = 130** |  |  |  |  | **H-,>A** |  |  |  | **H+,C-**  **n = 240** |  |  | **H+,≧A**  **n = 275** |
|  |  |  |  |  |  |  |  |  |  |  |  |  |  | **Bx** |  |  |  |
| **1y** |  |  |  |  | **PapH** |  |  |  |  |  |  |  |  |  |  |  |  |
|  |  |  |  |  |  |  |  |  |  |  |  |  |  |  |  |  |  |
|  |  |  | **H-,C-** | **H-,≧A** | **H+,≧A** | **H+,C-** |  |  |  |  |  |  |  |  |  |  |  |
|  |  |  |  |  | **Bx** |  |  |  |  |  |  |  |  |  |  |  |  |
| **2y** |  |  |  |  |  |  |  |  |  |  |  |  |  |  |  |  |  |
|  |  |  |  |  |  |  |  |  |  |  |  |  |  |  |  |  |  |
|  |  |  |  |  |  |  |  |  |  |  |  |  |  |  |  |  |  |
| **3y** | **Pap** |  |  |  |  |  |  |  |  |  |  |  |  |  |  |  |  |
| **4y** |  |  | **Pap** |  |  |  |  |  |  |  |  |  |  |  |  |  |  |

Rules: (1) baseline: any >A🡪 Bx

(2) throughout, any H+ 🡪 Bx

(3) 2 consecutive A, H+/- 🡪 Bx

H = HPV, C = cytology, A = Ascus, PapH = pap & HPV test, Bx = colposcopy +/- biopsy

Fig. 4 Intervention arm (post colposcopy – biopsy)

| **0** |  |  |  |  |  |  |  |  |  |  |  | **Bx** |  | Rules:   1. If 2 consecutive H-&C- 🡪 routine screening 2. Any H+ or C+ at / after 1^st^ y post Bx 🡪 repeat Bx   *CIN 1 in > 2 quad or persist for > 2yrs 🡪 Tx  Bx = colposcopy +/- biopsy  Tx = treatment  PapH = pap & HPV  H = HPV  C = Cytology  PapR = routine screening |
| --- | --- | --- | --- | --- | --- | --- | --- | --- | --- | --- | --- | --- | --- | --- |
|  |  |  |  |  |  |  |  |  |  | **≦CIN 1*** |  |  | **>CIN 1** |  |
|  |  |  |  |  |  |  |  |  |  |  |  |  | **Tx, Out** |  |
| **6m** | **post** |  |  |  |  |  |  |  | **Pap only** |  |  |  |  |  |
|  |  |  |  |  |  |  |  |  |  |  |  |  |  |  |
|  |  |  |  |  |  |  |  |  |  |  |  |  |  |  |
|  |  |  |  |  |  |  |  |  | **C- /A / LSIL** | **HSIL** |  |  |  |  |
|  |  |  |  |  |  |  |  |  |  |  |  |  |  |  |
|  |  |  |  |  |  |  |  |  |  | **Bx / Tx, Out** |  |  |  |  |
| **1y** | **post** |  |  |  |  |  |  |  | **PapH** |  |  |  |  |  |
|  |  |  |  |  |  |  |  |  |  |  |  |  |  |  |
|  |  |  |  |  |  |  |  |  |  |  |  |  |  |  |
|  |  |  |  |  |  | **H-,C-** | **H+,C-** |  | **A / LSIL / HSIL (H+/-)** |  |  |  |  |  |
|  |  |  |  |  |  |  |  |  |  |  |  |  |  |  |
|  |  |  |  |  |  |  |  |  | **Bx** |  |  |  |  |  |
| **2y** | **post** |  |  |  |  | **PapH** |  |  |  |  |  |  |  |  |
|  |  |  |  |  |  |  |  |  |  |  |  |  |  |  |
|  |  |  |  |  |  | **H-,C-** |  |  | **Any H+ or C+** |  |  |  |  |  |
|  |  |  |  |  |  |  |  |  |  |  |  |  |  |  |
|  |  |  |  |  |  |  |  |  | **Bx** |  |  |  |  |  |
| **5y** | **post** |  |  |  |  | **PapR** |  |  |  |  |  |  |  |  |
|  |  |  |  |  |  |  |  |  |  |  |  |  |  |  |
|  |  |  |  |  |  |  |  |  |  |  |  |  |  |  |
